# The Supplementation of *Sechium edule* var.* nigrum spinosum* (Chayote) Promotes Nrf2-Mediated Antioxidant Protection in Older Adults with Metabolic Syndrome

**DOI:** 10.3390/nu15194106

**Published:** 2023-09-22

**Authors:** Graciela Gavia-García, David Hernández-Álvarez, Taide Laurita Arista-Ugalde, Itzen Aguiñiga-Sánchez, Edelmiro Santiago-Osorio, Víctor Manuel Mendoza-Núñez, Juana Rosado-Pérez

**Affiliations:** 1 Research Unit on Gerontology, FES Zaragoza, National Autonomous University of Mexico, Mexico City 09230, Mexico; graciela_gavia_garcia@comunidad.unam.mx (G.G.-G.); hereda2912@gmail.com (D.H.-Á.); tdlarista@comunidad.unam.mx (T.L.A.-U.); 2 Hematopoiesis and Leukemia Laboratory, Research Unit on Cell Differentiation and Cancer, FES Zaragoza, National Autonomous University of Mexico, Mexico City 09230, Mexico; itzen.aguiniga@zaragoza.unam.mx (I.A.-S.); edelmiro@unam.mx (E.S.-O.)

**Keywords:** *Sechium edule*, metabolic syndrome, antioxidants, inflammatory markers, aging, mRNA

## Abstract

The aim was to determine the effect of *Sechium edule* var. *nigrum spinosum* (chayote) on gene expression related to antioxidant protection mechanisms and the inflammatory process in older adults with metabolic syndrome (MetS). A quasi-experimental study was carried out in a convenience sample of 46 older adults diagnosed with MetS: (i) placebo group (PG; n = 20); (ii) experimental group (EG; n = 26). The clinical, biochemical, anthropometric parameters and SOD, GPx, and CAT enzyme activity, alongside total oxidant status (TOS), total antioxidant status (TAS), oxidative stress index (OSI), cytokines (IL-6, IL-8 and TNF-α), and mRNA expression of SOD, GPx, CAT, IL-6, IL-8, TNF-α, Nrf2, NFkB p50, and NFkB p65, were measured at baseline and 6 months post-intervention. A statistically significant decrease was observed in TOS (baseline, 28.9 ± 3.6 vs. post, 23.7 ± 3.4, *p* < 0.01) and OSI (baseline, 24.1 ± 3.8 vs. post, 17.7 ± 4), as well as an increase in IL-6 (baseline, 10.7 ± 1.1 vs. post, 12.3 ± 2, *p* = 0.03), SOD activity (baseline, 167.1 ± 11.9 vs. post, 180.6 ± 7.6, *p* < 0.05), CAT activity (baseline, 1.0 ± 0.2 vs. post, 1.3 ± 0.2, *p* < 0.01), and TAS (baseline, 1.1 ± 0.1 vs. post, 1.4 ± 0.1, *p* < 0.01) in the EG compared to the PG. Regarding the expression of Nrf2, SOD, and IL-6, the EG showed a significant increase vs. basal levels (47%, 44%, and 43%, respectively). Our findings suggest that *Sechium edule* supplementation promotes the antioxidant response and decreases oxidative stress via Nrf2.

## 1. Introduction

In recent years, there has been an increase in the number of older adults worldwide; projections estimate that by 2030, 16% of the population will be 60 years of age or older [1]. In this sense, human aging is the result of a gradual and adaptive process characterized by a decrease in the biological response accompanied by social, psychological, morphological, physiological, and biochemical modifications, favored by the genetic load and accumulated wear and tear in the face of the challenges that a person faces over time throughout his or her history [2]. Aging can predispose a person to the development of non-communicable chronic diseases, among them metabolic syndrome (MetS).

MetS leads to high rates of morbidity and mortality and represents a major threat to public health systems since it increases the risk of presenting cardiovascular disease, type II diabetes mellitus (T2DM), development of neurodegenerative diseases, and ultimately death [3,4,5]. In this respect, recent evidence shows that systemic inflammation derived from the intense endocrine activity of adipose tissue in obesity is a risk factor for depression, reduction in brain dimensions, brain injuries, and cognitive activity, as well as the progression of cardiovascular diseases such as myocarditis, heart failure, systemic heart disease and strokes, altered lipid metabolism, and diabetes complications. Molecules such as nuclear factor kappa B (NFκB), peroxisome proliferator-activated receptor a (PPARa) and brain-derived neurotrophic factor (BDNF) have been identified as central actors in this process through various mechanisms [6,7,8,9,10,11,12].

The MetS prevalence in Latin America is almost 25%, with women and older adults being the most compromised [13]. Metabolic abnormalities that accompany this syndrome include at least three of the following disorders: (i) arterial hypertension, (ii) increased blood glucose, (iii) central (abdominal) obesity, and (iv) dyslipidemia [increased triglycerides and decreased high-density lipoprotein cholestaerol (HDL-c)] [14].

Oxidative stress (OxS) and chronic inflammation (CI) play a significative role in the pathogenesis of MetS [4,15]. The increase in reactive oxygen species (ROS) in MetS is pointed out as an underlying mechanism of mitochondrial dysfunction and accumulation of oxidative damage to proteins, lipids, and nucleic acids, a consequence of a poor antioxidant status compared to those who do not suffer MetS [16,17,18].

Likewise, various studies have shown that MetS is associated with a condition of low-grade inflammation, denoted by atypical production of proinflammatory cytokines and acute phase reactants such as interleukin-6 (IL-6), tumor necrosis factor alpha (TNF-α), fibrinogen, and C reactive protein (CRP).

It has been pointed out that individually each of the MetS alterations by themselves are associated with both OxS and chronic inflammation [19]. Hence, the importance of finding strategies to avoid the complications that the presence of MetS entails; pharmacotherapy has been proposed, as well as the implementation of healthy lifestyle practices such as regular physical activity and a healthy diet [17,18,20]. However, adopting healthy habits is usually difficult for the population, so it is still necessary to explore new strategies such as nutraceutical supplements, which are well accepted among the elderly.

In this regard, *Sechium edule* (chayote) is a fruit fit for human consumption of the *Cucurbitaceae* family, in the varietal group *nigrum spinosum*, which presents high quality in its nutritional characteristics given that its protein content is 1.20%, with 1.96% crude fiber, 8.49% assimilable carbohydrates, and 0.08% crude fat (g of component/100 g of sample on wet basis). Likewise, chayote has seven of the nine essential amino acids, and its fat content is less than 1%. Hence, it is widely recommended in diets with low-calorie content and in the meal plans of hospital diets [21]. In addition, various benefits for human health have been attributed due to its antioxidant, anti-inflammatory, antitumor, and hypotensive properties [22,23,24]. Our research team has shown that consumption of *Sechium edule* reduces OxS and IC related to MetS and [25]. However, the transcripts and regulators that could be involved in this decrease are still unknown. In this sense, the Kelch-like ECH-associated protein 1 (KEAP1)/nuclear factor erythroid 2-related factor 2 (Nrf2) pathway is of interest due to its pleiotropic nature and its cytoprotective effects as a consequence of the activation of genes with antioxidant function, but it can also lead to the appearance, progression, and survival of cancer cells if they are hyperactivated, as has been observed in several types of cancer [26,27,28,29]. Hence the relevance of the present study, whose objective was to determine the effect of *Sechium edule* supplementation on gene expression related to antioxidant protection mechanisms and the inflammatory process in older adults with MetS.

## 2. Materials and Methods

### 2.1. Experimental Design

This study was approved by the Bioethics in Research and Biosafety Committee of the Faculty of Higher Studies Zaragoza UNAM (23/02-SO/2.4.2) with trial registration number (ISRCTN: 43215432). Informed consent was obtained from each of the participants. The procedures were carried out respecting the ethical codes of the Declaration of Helsinki of the World Medical Association. The fruits of *Sechium edule* varietal group *nigrum spinosum* used in the study were donated by the Interdisciplinary Research Group at *Sechium edule* A.C. (GISeM) of the RED-Chayote, of the Agricultural Genetic Resources Subcommittee of the National Seed Inspection and Certification Service (NSICS), which has worked to characterize, preserve, improve, and enhance the genus *Sechium* in Mexico and has established the Germplasm Bank of *Sechium edule* in Mexico in the Municipality of Huatusco in the State of Veracruz, where the fruits used to make the capsules used in the study are collected [30]. The characterization of the fruits was carried out according to the guidelines proposed by the International Union for the Protection of New Varieties of Plants (UPOV) with the validation of descriptors such as morphological, chromosomal, and phenotypic characterization, which was performed using GISeM [21,30]. The biological material was collected in a state of horticultural maturity; it was selected and subsequently washed, partitioned into slices, dried at 40 °C, and pulverized (epidermis, seeds, and spines). The formulation of the capsules, both active and placebo, was carried out in the pharmaceutical development laboratory of the FES Zaragoza. The placebo was made with lactose monohydrate and talc, both pharmaceutical grade (United States Pharmacopeia, USP) (Sigma, St. Louis, MO, USA). The optimal particle size of the *Sechium* powder was determined, and rheological studies were carried out to guarantee the homogeneity, filling, and stability of the capsules. After the design, the treatments were prepared and packaged by a pharmaceutical company specializing in nutraceutical products.

### 2.2. Intervention

The intervention consisted of consuming three 500 mg/day capsules of *Sechium edule* or placebo (one before each meal) for six months. The selection of the dose according to the phytochemical and safety profile has already been described by our work group [25,31]. The type of sampling was for convenience in a population of 46 older adults, with an average age of 67.6 ± 5.85. All participants were diagnosed with MetS according to the National Cholesterol Education Program Adult Treatment Panel (NCEP/ATP III) criteria: (i) waist circumference ≥ 102 cm for men or ≥88 cm for women; (ii) blood pressure ≥ 130/85 mmHg; (iii) glucose > 110 mg/dL; (iv) high-density lipoprotein cholesterol (HDL-c)  <  40 mg/dL in men or <50 mg/dL in women; and (v) triglycerides ≥ 150 mg/dL. MetS was diagnosed with presence of at least three of five criteria [32].

Participants were randomly designated to the experimental group with *Sechium edule* intervention (EG; n = 50) or to the placebo group (PG; n = 50). Of these patients, only certain participants wished to donate venous blood for gene expression determinations (PG, n = 20; EG, n = 26). In all groups, all measurements were made before receiving treatment (baseline measurements) and six months later (post-treatment measurements).

### 2.3. Anthropometric and Clinical Measurements

Body weight and waist circumference were determined. Body weight was measured with a calibrated medical scale (SECA, Hamburg, Germany), whereas the waist circumference was measured at the level of the navel with a medical tape (SECA, Hamburg, Germany). Trained personnel from the FES Zaragoza were responsible for the aforementioned measurements [33].

To determinate systolic (SBP) and diastolic (DBP) blood pressure, a calibrated mercury Baumanometer was used. The patient remained at rest before taking the blood pressure (five minutes at least), sitting in a seat with a backrest, with a straight back, feet resting on the floor and legs uncrossed. To identify pseudohypertension, the Osler technique was used [34].

### 2.4. Biochemical Analysis

#### 2.4.1. Samples

After fasting for 8 h, blood samples were obtained with venipuncture, collected in vacuum tubes without anticoagulant for clinical chemistry determinations (glucose, triglycerides, and high-density lipoprotein cholesterol (HDL-c)) and serum cytokines; in tubes with EDTA as anticoagulant for the determination of catalase activity (CAT) and extraction of lymphocytes; and in tubes with sodium heparin for the determination of erythrocyte superoxide dismutase (SOD) and glutathione peroxidase (GPx) activity. Meanwhile, total antioxidant status (TAS) and total oxidant status (TOS) were determined in heparinized plasma. The SOD, GPx, CAT, TAS, and TOS techniques were performed at microscale in multi-well plates, which were read on a Multiskan Go spectrophotometer, version 1.00.40 (Thermo Scientific, Denver, CO, USA). The lipid and glycemic profiles were determined using spectrophotometric techniques with an automated clinical chemistry analyzer Selectra Junior (Vital Scientific, Dieren, The Netherlands).

#### 2.4.2. SOD Enzyme Activity

The activity of the SOD enzyme was determined using a commercial kit (Randox Laboratories Ltd., Antrim, UK. Cat. SD125), according to the supplier’s instructions. The test is based on the generation of the superoxide radical (by xanthine oxidase) and its subsequent reaction with 2-(4-iodophenyl)-3-(4-nitrophenol)-5-phenyl-tetrazolium chloride to form the formazan dye, which is read at 505 nm. The inhibition of this reaction in the presence of SOD allows us to determine its activity.

#### 2.4.3. GPx Enzyme Activity

In the presence nicotinamide adenine dinucleotide phosphate (NADPH) and glutathione reductase, glutathione peroxidase (GPx) catalyzes the oxidation of glutathione (GSH) by cumene hydroperoxide. Oxidized glutathione (GSSG) is immediately converted into the reduced form with the subsequent oxidation of NADPH to NADP (Randox Laboratories, Ltd., Crumlin Co., Crumlin, UK. Cat. RA504). The decrease in absorbance was measured at 340 nm UV-spectrophotometer with a Multiskan TM Go Microplate (Thermo Scientific TM).

#### 2.4.4. Catalase Activity

The catalase activity was determined with spectrophotometry, using hydrogen peroxide (H_2_O_2_) (Sigma, St. Louis, MO, USA) as substrate. For this, a mixture of 190 μL of working solution (0.1 M phosphate buffer, pH  =  7.0 and 20 mM H_2_O_2_) and 10 μL of the sample were made. The decrease in H_2_O_2_ concentration was measured every 15 s for 3 min at 240 nm [35].

#### 2.4.5. Total Oxidant Status (TOS)

Plasma TOS determination was performed employing a commercial kit (Rel Assay Diagnostics, Gaziantep, TR), following the provider’s instructions. Oxidants present in the sample oxidize the ferrous ion-chelating complex to ferric ion. The latter forms a color complex with the chromogen in an acid medium in such a way that the intensity of the color is directly proportional to the amount of oxidant molecules present in the sample. Measurements were made at a wavelength of 530 nm.

#### 2.4.6. Total Antioxidant Status (TAS)

Total antioxidant status was determined using a commercial kit (Randox Laboratories Ltd., Antrim, UK. Cat. NX2332) based on the reaction of 2,2′-azino-bis (3-ethylbenzthiazoline-6-sulfonic acid) (ABTS) with metmyoglobin and H_2_O_2_. This reaction stimulates the formation of the cationic radical ABTS^+^, which produces a blue-green color. The intensity of the color is inversely proportional to quantity of antioxidants existent in the sample. The reaction was measured at 600 nm.

#### 2.4.7. Inflammatory Cytokines

Serum levels of interleukin-6 (IL-6), interleukin-8 (IL-8), and tumor necrosis factor alpha (TNF-α) were determined using the Human Inflammatory Cytokine Cytometric Bead Array (CBA) Kit (BD, Biosciences, San Jose, CA, USA. Cat. 551811), which involves a sandwich capture method with beads conjugated with specific antibodies and a detection reagent (phycoerythrin mixture-conjugated antibodies). This results in a fluorescent signal in proportion to the number of bound analytes, which were detected using a flow cytometer (BD Biosciences, San Jose, CA, USA) and the FCAP ArrayTM version 3.0 software.

#### 2.4.8. Lymphocyte Isolation and RNA Extraction

Lymphocyte isolation was performed from 5 mL of venous blood, diluted 1:1 with phosphate buffered saline (PBS) (Sigma, St. Louis, MO, USA)/2% fetal bovine serum (FBS) (ThermoFisher Scientific, Waltham, MA, USA). Subsequently, 4 mL of Ficoll-Paque (Gibco ThermoFisher Scientific, Waltham, MA, USA) were added. It was centrifuged at 200× *g*, and the opaque interface of interest was removed. From 2 × 10^6^ lymphocytes, RNA extraction was performed using an RNeasy Mini kit isolation kit (Qiagen, Hilden, Düsseldorf, Germany), following the manufacturer’s recommendations. RNA extraction was performed just after lymphocyte isolation, aliquoted, and stored at −70 °C until further use. The RNA was quantified, and its integrity was determined from 5 µg in a 1% agarose gel with ethidium bromide and tris-acetate-EDTA buffer visualized in a Kodak Molecular Imaging Software (v.4.5.1) imaging system, while its purity was calculated using the A260/A280 ratio. All the RNA samples used in the present study were considered intact and with an optimal purity value.

#### 2.4.9. Gene Expression Analysis

The reactions were performed from 10 ng of RNA and forward and reverse primers at a final concentration of 100 nM (IDT, Coralville, IA, USA) (Table 1). It should be noted that the primers were designed with the NCBI Primer-BLAST tool from NIH [36]. For the analysis of gene expression, the QuantiFast SYBR Green RT-PCR kit (one-step RT-PCR) kit (Qiagen, Hilden, Düsseldorf, Germany) was used, which allows the simultaneous execution of the reverse transcription and the PCR reaction. Reaction conditions were as follows: 50 °C for 10 min for reverse transcription, 95 °C for 5 min for PCR initial activation step, followed by 40 three-segment cycles to amplify the specific PCR product: denaturation at 95 °C for 10 s, annealing at 60 °C for 30 s, and extension at 72 °C for 15 s. The mean crossing threshold (Ct) of each gene was normalized to the mean Ct of the housekeeping gene β-actin.

## 3. Results

Table 2 shows the anthropometric and clinical markers of both study groups: experimental group (EG) and placebo group (PG), pre- and post-treatment. The EG showed a statistically significant decrease in both SBP (baseline, 141.4 ± 12.1 vs. post, 131.2 ± 11.2, *p* < 0.01) and DBP (baseline, 95 ± 9.8 vs. post, 83.8 ± 8, *p* < 0.001), as well as in body weight at six months post-treatment (baseline, 74.4 ± 16.5 vs. post, 72.4 ± 16.4, *p* < 0.01). On the other hand, no statistically significant changes were observed in waist circumference regardless of the intervention.

Concerning the biochemical parameters related to the pathophysiology of MetS, only the concentration of HDL-c in the EG increased at six months post-treatment (baseline, 42.8 ± 7.5 vs. post, 47.4 ± 7.4, *p* < 0.01) compared to the PG. Meanwhile, glucose and triglyceride levels did not show apparent changes in either post-treatment groups (Table 3).

Regarding the antioxidant activity markers, a statistically significant increase was observed in the post-treatment in the EG compared to the PG in the activity of enzymes with antioxidant function, SOD (baseline, 167.1 ± 11.9 vs. post, 180.6 ± 7.6, *p* < 0.05) and CAT (baseline, 1.0 ± 0.2 vs. post, 1.3 ± 0.2, *p* < 0.01), as well as in the total antioxidant capacity (TAS) (baseline, 1.1 ± 0.1 vs. post, 1.4 ± 0.1, *p* < 0.01); on the contrary, there was a decrease in the total oxidant status (TOS) (baseline, 28.9 ± 3.6 vs. post, 23.7 ± 3.4, *p* < 0.01) and in the OSI (EG: baseline, 24.1 ± 3.8 vs. post, 17.7 ± 4 vs. PG: baseline, 19.2 ± 2.1 vs. post, 26.1 ± 7, *p* < 0.01) in the EG (Table 4).

Regarding the interleukins, a statistically significant increase was observed in the IL-6 concentration of the EG post-treatment (baseline, 10.7 ± 1.1 vs. post, 12.3 ± 2, *p* = 0.03), compared to the PG. The levels of IL-8 and TNF-α did not present statistically significant changes (Table 5).

**Figure 1:**
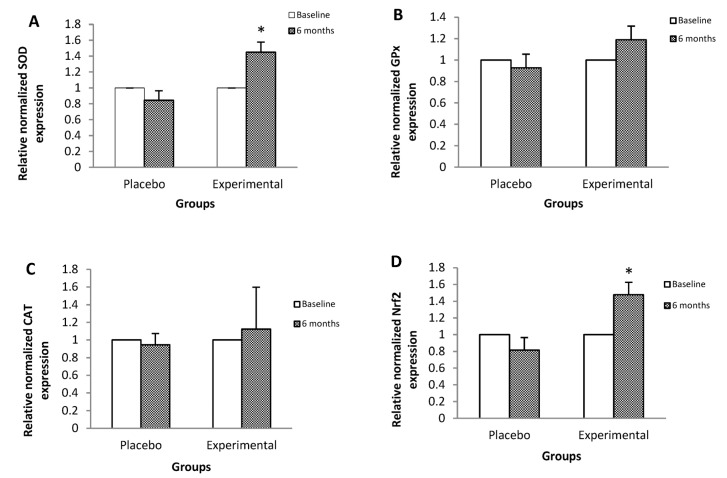

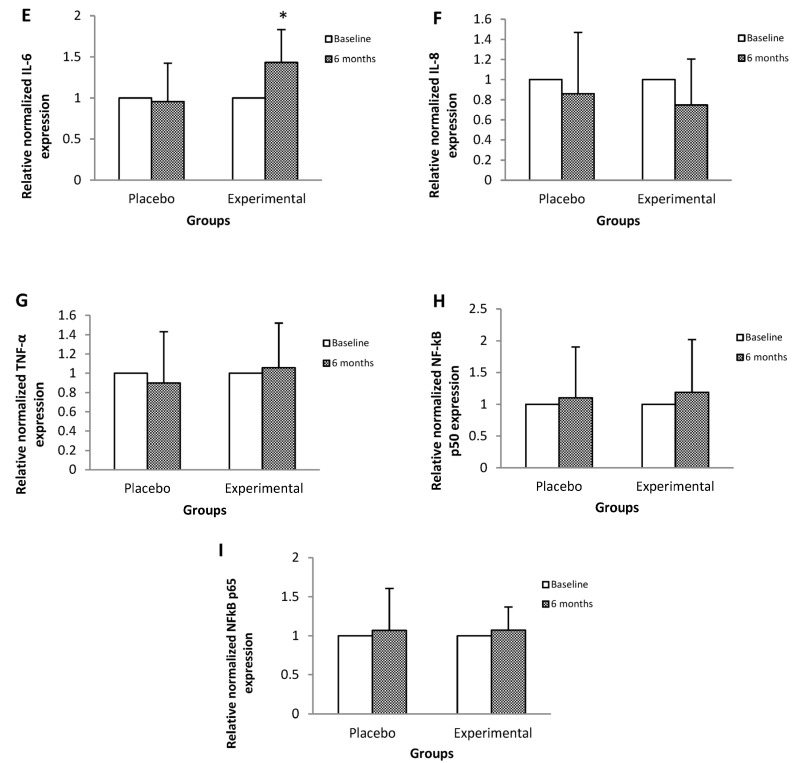
Relative expression of the mRNA of genes that code for proteins that participate in processes of antioxidant protection and inflammation, among the study subjects. Data are expressed as means ± standard deviation. ANOVA of repeated measures test, significance level 95%, *p* < 0.05. (**A**) SOD, superoxide dismutase; (**B**) GPx, glutathione peroxidase; (**C**) CAT, catalase; (**D**) Nrf2, nuclear factor (erythroid-derived 2)-like 2; (**E**) IL-6, interleukin-6; (**F**) IL-8, interleukin-8; (**G**) TNF-α, tumor necrosis factor-alpha; (**H**) NFκB p50, nuclear factor kappa B p50; (**I**) NFκB p65, nuclear factor kappa B p65. The mRNA relative expression levels were determined after normalization against β-actin. * Baseline statistical significance vs. 6 months inter-group.

Figure 1 shows the relative expression of the mRNA of genes that code for proteins that participate in antioxidant protection mechanisms and in the inflammatory process. SOD (Figure 1A), Nrf2 (Figure 1D), and IL-6 (Figure 1E) gene expressions showed a statistically significant increase in post-treatment EG of 44%, 47%, and 43%, respectively, with respect to their basal levels.

However, there were no significant changes in the mRNA of GPx (Figure 1B), CAT (Figure 1C), IL-8 (Figure 1F), TNF-α (Figure 1G), NFκB p50 (Figure 1H), or p65 NFκB (Figure 1I).

## 4. Discussion

MetS is an ensemble of systemic amendments whose pathophysiology has been linked to a pro-oxidative and proinflammatory state [14]. It has been pointed out that, individually, each of the MetS alterations is linked to OxS [32]. On the other hand, various studies have shown that MetS is related to a condition of low-grade inflammation, branded by anomalous generation of proinflammatory cytokines and acute phase reactants such as TNF-α, fibrinogen, and CRP. Given the high prevalence of this syndrome, and the difficulty of treating the set of alterations that compose it, it is necessary to find alternatives that improve its control. In this sense, *Sechium edule* has been shown to have hypotensive, lipolytic, antioxidant, and anti-inflammatory effects, which makes it a feasible option for older adults in whom there is also a greater acceptance of natural treatments [37].

The results of this research reveal that the ingesting of *Sechium edule* has positive effects at biochemical and systemic levels. On the one hand, we observed that it promoted weight loss in the study population, a finding consistent with previous results reported by our research team, which can be supported by its lipolytic effect. It has been reported that the polyphenols present in *Sechium edule* activate signaling pathways that decrease the activity of lipogenic relative enzymes, such as FAS (fatty acid synthase), HMGCoR (HMG-CoA reductase), and SREBPs (sterol regulatory element binding proteins), and increase the expression of CPT-I (carnitine palmitoyltransferase I) and PPARα (peroxisome proliferators activated receptor α), which are critical regulators of hepatic lipid metabolism [38,39], and this is associated with the direct effect of *Sechium edule* on the digestive process due to its low caloric content and high amount of insoluble fiber that allows weight loss, which is a significant result given the role of obesity in the pathophysiology of high-frequency, non-communicable chronic diseases among this population group.

Likewise, regarding blood pressure, we observed a significant decrease, a finding that may be due to the activity of flavonoids, such as quercetin and coumaric acid, which affect the renin-angiotensin system and the modulation of calcium release with an effect on endothelial vasodilation and consequently on blood pressure; this result is also consistent with previous findings reported by our research group [24,25,31].

With regard to circulating lipids, a significant increase in HDL-c was observed in the group that consumed *Sechium edule* in this investigation. In this regard, it has been pointed out that compounds such as naringenin and quercetin influence HDL function beyond HDL-c concentration by regulating cellular cholesterol efflux from macrophages and hepatic paraoxonase 1 expression and activity [40].

On the other hand, for the determined OxS markers, it was observed that the consumption of *Sechium edule* had a marked antioxidant effect, a finding that coincides with and corroborates the previous results of our research group. In this case, we found a significant increase in the activity of the SOD and CAT enzymes, as well as an increase in TAS, a marker that reflects extracellular antioxidant activity. Likewise, there was a decrease in the TOS oxidation marker coupled with a decrease in OSI, an index that reflects the TOS/TAS relationship; together, these results disclose that the group that consumed *Sechium edule* had an increase in antioxidant activity with a concomitant decrease in oxidation. Likewise, an effect on IL-6 was observed, which increased significantly in the EG, which may suggest an impact on the inflammatory process, which is closely related to OxS, since the regulatory mechanisms frequently present intersection points. These effects, as we have pointed out in detail in a previous investigation [31], have been consistently reported and are due to the variety of bioactive compounds, specifically, phenolic acids, cucurbitacins, and flavonoids present in chayote capsules, which synergistically affect the activity and even expression of antioxidant molecules.

Regarding the mechanisms behind this effect, there is evidence of the signaling pathways in which these bioactive compounds interfere; in various in vitro and in vivo studies, the capacity of some of these molecules, for example, quercetin and naringenin, has been demonstrated to bind to the master regulator of the antioxidant response Nrf2 to DNA [41,42,43].

Concerning cucurbitacins, their chemoprotective and antioxidant capacity has been shown [44,45,46]. Specifically, cucurbitacins I, D, B, and E can promote the expression of heme oxygenase-1 (HO-1) and NAD(P)H dehydrogenase quinone 1 (NQO-1), which are phase II detoxification enzymes; the mechanisms proposed implies the regulation of the Nrf2 transcription factor, which in turn modulates the expression of SOD, GPx, and CAT. Also, cucurbitacins promote the inhibition of nuclear factor, increasing the kappa light chain of activated B cells (NFκB) [47].

Also, myricetin, a flavonoid, has demonstrated a protective effect since it increases the nuclear accumulation of Nrf2 and blocks NFκB [48,49]. In addition, phlorizin promotes the translocation of Nrf2 to the nucleus and upregulates its downstream antioxidant response element (ARE), which contains the enzymes HO-1 and NQO-1 in an animal model with oxidative harm [50]. Furthermore, naringenin has the ability to reduce ROS levels through the Nrf2/ARE pathway [51]. In terms of the phenolic acids existing in *Sechium edule*, it has been pointed out that they present antioxidant and anti-inflammatory effects through Nrf2 and NFκB, respectively. With respect to chlorogenic acid, it is reported to have a protective effect on the nephron through the inhibition of OxS and inflammatory processes [52]; regarding gallic acid, it has been shown that it increases nuclear Nrf2 levels, thereby reducing oxidative injury [53].

These findings are consistent with those previously reported by our research group and are consistent with the results obtained in this work regarding the expression of the genes of the molecules involved in the antioxidant response.

In this sense, it has been shown in experimental models of aging that the expression of genes that code for proteins that participate in antioxidant defense mechanisms decreases, suggesting that this alteration is involved in oxidative damage, possibly due to a decrease in cell signaling [54,55,56].

In the present study, we were able to observe a parallel increase in the relative expression levels of SOD mRNA by around 12% and enzymatic activity by 8% in the post-treatment EG. That is, the consumption of *Sechium edule* improves the efficiency of the SOD enzyme from transcriptional levels, which makes the superoxide anion less dangerous for the cell [44], leading to a reduction in oxidative damage in lipids, proteins, and DNA, as previously reported [25,31,57,58,59].

On the other hand, we observed that gene expression and GPx activity did not present statistically significant changes in the study groups. Meanwhile, CAT activity increased by 30%, without significant changes in its mRNA levels; these results lead us to assume that the increase in SOD and plasmatic antioxidants (TAS) were sufficient to counteract the reactive species, so that there was no increase in the de novo synthesis of CAT and nor in that of GPx because it was not necessary [54,60].

In terms of the expression and content of IL-6, it increased by 43% and 20%, respectively, in the post-treatment EG. Regarding this cytokine, a possible duality has been pointed out depending on the conditions of the organism; it has been reported that, in pathologies such as T2DM, there is a loss of IL-6 signaling leading to oxidative damage and cell death [61]. At the same time, the increase in its levels protects from this type of damage at the DNA level, coupled with a positive regulation of SOD via Nrf2, which agrees with our results [62]. Therefore, our findings suggest that, in this case, IL-6 exerts an antioxidant-like protective effect against OxS mediated by Nrf2 [63].

In this sense, in the present study, an increase in Nrf2 mRNA levels was observed by 47% in the post-treatment EG, compared to its basal levels, which is consistent with the overall increase in the antioxidant response observed.

Our results show there were no significant changes in the mRNAs of the NFκB p50/p65 factor. This allows us to assume that the transcriptional regulation of genes involved in the inflammatory process is more difficult to modify through nutraceutical supplementation.

Finally, it is worth mentioning that, as explained in detail in a previous study, practically all the secondary metabolites existing in *Sechium edule* modulate the expression of Nrf2 mRNA; for example, cucurbitacins, naringenin, and caffeic and ferulic acids have been related to an increase in Nrf2 gene expression and inhibition of NFκB-mediated signaling [64,65,66,67]. Apigenin, myricetin, and gallic acid improve translocation and/or nuclear accumulation [68,69,70]. Quercetin only temporarily stimulates its expression [71]. Phlorizin, rutin, and chlorogenic and protocatechuic acids enhance antioxidant protection through this same pathway [72,73,74,75], and finally the hypolipidemic effects exerted by p-coumaric acid are directly related to the expression of Nrf2 [76].

## 5. Conclusions

The results of our research, that is, the increase in the levels of gene expression, activity, or content of SOD, CAT, and IL-6, as well as the expression of Nrf2, in the group that consumed *Sechium edule*, suggest that the globally observed antioxidant effect is mediated by the transcriptional factor Nrf2. Hence, some of the perspectives of this line of research are to determine if there is a relationship between the consumption of this fruit and some Nrf2 coactivators such as sirtuins and, therefore, to determine if there is a relationship with the adaptation of metabolic alterations.

## Figures and Tables

**Table 1:** Detailed primers used for real-time PCR assays.

Gene	Primer Name	Primer Sequence
*SOD1*	SOD-F	GGTGGGCCAAAGGATGAAGA
	SOD-R	ATAGACACATCGGCCACACC
*GPX1*	GPX-F	ACACCCAGATGAACGAGCTG
	GPX-R	CTTCGTTCTTGGCGTTCTCC
*CAT*	CAT-F	TGAAGATGCGGCGAGACTTT
	CAT-R	GAGGGGTACTTTCCTGTGGC
*NFE2L2*	NRF2-F	AGGTTGCCCACATTCCCAAA
	NRF2-R	ACGTAGCCGAAGAAACCTCA
*IL-6*	IL6-F	CCACCGGGAACGAAAGAGAA
	IL6-R	GAGAAGGCAACTGGACCGAA
*IL-8*	IL8-F	GAAGAGAGCTCTGTCTGGACC
	IL8-R	TGAATTCTCAGCCCTCTTCAAAAAC
*TNF-α*	TNF-F	GAAGAGAGCTCTGTCTGGACC
	TNF-R	TGAATTCTCAGCCCTCTTCAAAAAC
*NFKB-p50*	NFKBP50-F	GGAGGCCGAACGCGG
	NFKBP50-R	AAACATTTGTTCAGGCCTTCCC
*NFKB-p65*	NFKB65-F	CAGTGTGTGAAGAAGCGGGA
	NFKB65-R	CCACGCTGCTCTTCTTGGAA
*β-ACTIN*	ACTIN-F	GAGCACAGAGCCTCGCC
	ACTIN-R	CGCGGCGATATCATCATCCA

SOD1, copper/zinc-superoxide dismutase; GPX1, glutathione peroxidase 1; CAT, catalase; NFE2L2, nuclear factor, erythroid 2-like 2; IL-6, interleukin 6; IL-8, interleukin 8; TNF-α, tumor necrosis factor-alpha; NFκB-p50, nuclear factor kappa B subunit-p50; NFκB-p65, nuclear factor kappa B subunit-p65.

**Table 2:** Clinical and anthropometric measurements by study group.

Parameter	Placebo n = 20	Experimenta ln = 26	*p*-Value
Age (years)	68.4 ± 5.7	67.9 ± 6.6	
SBP (mmHg)			
Baseline	132.5 ± 15.5	141.4 ± 12.1	
6 months	132.3 ± 12.1	131.2 ± 11.2 *	0.01
DBP (mmHg)			
Baseline	90.2 ± 9.9	95.0 ± 9.8	
6 months	85.6 ± 6.4	83.8 ± 8.0 *	0.001
Weight (kg)			
Baseline	76.5 ± 14.6	74.4 ± 16.5	
6 months	76.2 ± 12.8	72.4 ± 16.4 *	0.01
Waist circumference (cm)			
Baseline	103.6 ± 11.4	99.9 ± 12.2	
6 months	104.2 ± 11.9	102.1 ± 12.1	0.55

The data are expressed as the average ± standard deviation. ANOVA of repeated measures, significance level 95%, *p* < 0.05. SBP, systolic blood pressure; DBP, diastolic arterial pressure. * Baseline statistical significance vs. 6 months inter-group.

**Table 3:** Pre- and post-treatment biochemical parameters by study group.

Parameter	Placebo n = 20	Experimental n = 26	*p*-Value
Glucose (mg/dL)			
Baseline	137.3 ± 61.3	140.7 ± 48.1	
6 months	139.4 ± 69.1	140.0 ± 57.3	0.91
HDL-c (mg/dL)			
Baseline	47.8 ± 9.5	42.8 ± 7.5	
6 months	47.4 ± 7.5	47.4 ± 7.4 *	0.01
Triglycerides (mg/dL)			
Baseline	169.6 ± 36.6	174.1 ± 54.8	
6 months	125.7 ± 30.4	149.2 ± 45.6	0.19

The data are expressed as the mean ± standard deviation. ANOVA of repeated measures, significance level 95%, *p* < 0.05. HDL-c, high-density lipoprotein cholesterol. * Baseline statistical significance vs. 6 months inter-group.

**Table 4:** Antioxidant capacity and oxidative state by study group.

Parameter	Placebo n = 20	Experimental n = 26	*p*-Value
SOD (U/mL)			
Baseline	176.8 ± 10.3	167.1 ± 11.9	
6 months	174.6 ± 4.9	180.6 ± 7.6 *	0.05
GPx (U/L)			
Baseline	5740 ± 938	5221 ± 822	
6 months	5023 ± 1885	5559 ± 2007	0.33
CAT (U/mL)			
Baseline	1.2 ± 0.1	1.0 ± 0.2	
6 months	1.2 ± 0.2	1.3 ± 0.2 *	0.01
TOS (µmol H_2_O_2_ Equiv./L)			
Baseline	24.5 ± 2.3	28.9 ± 3.6	
6 months	28.0 ± 3.7	23.7 ± 3.4 *	0.01
TAS (mmol/L)			
Baseline	1.2 ± 0.1	1.1 ± 0.1	
6 months	1.1 ± 0.2	1.4 ± 0.1 *	0.01
OSI			
Baseline	19.2 ± 2.1	24.1 ± 3.8	
6 months	26.1 ± 7.0	17.7 ± 4.0 *	0.01

The data are expressed as the mean ± standard deviation. ANOVA of repeated measures, significance level 95%, *p* < 0.05. SOD, superoxide dismutase; GPx, glutathione peroxidase; CAT, catalase; TOS, total oxidizing state; TAS, total antioxidant status; OSI, oxidative stress index (TOS/TAS). * Baseline statistical significance vs. 6 months inter-group.

**Table 5:** Pre and post-treatment inflammatory markers by study group.

Parameter	Placebo n = 20	Experimental n = 26	*p*-Value
IL-6 (pg/dL)			
Baseline	10.7 ± 2.1	10.7 ± 1.1	
6 months	11.0 ± 0.9	12.3 ± 2.0 *	0.03
IL-8 (pg/dL)			
Baseline	27.6 ± 4.4	37.7 ± 9.9	
6 months	25.8 ± 6.0	30.9 ± 11.1	0.12
TNF-α (pg/dL)			
Baseline	8.4 ± 1.2	8.2 ± 0.6	
6 months	8.8 ± 1.6	9.2 ± 1.1	0.61

Data are expressed as means ± standard deviation. ANOVA of repeated measures test, significance level 95%, *p* < 0.05. IL-6, interleukin-6; IL-8, interleukin-8; TNF-α, tumor necrosis factor-alpha. * Baseline statistical significance vs. 6 months inter-group.

## Data Availability

The data that support the findings of this study are available from the corresponding author, [V.M.M.-N.], upon reasonable request.
